# Evaluation of the efficacy of memantine in the treatment of fibromyalgia: study protocol for a doubled-blind randomized controlled trial with six-month follow-up

**DOI:** 10.1186/1745-6215-14-3

**Published:** 2013-01-03

**Authors:** Bárbara Olivan-Blázquez, Marta Puebla, Bárbara Masluk, Mari-Cruz Pérez-Yus, Raquel Arcega, Eva Andrés, Yolanda López-del-Hoyo, Rosa Magallon, Miquel Roca, Javier Garcia-Campayo

**Affiliations:** 1 Department of Psychology and Sociology, University of Zaragoza, Zaragoza, Spain; 2 Red de Actividades Preventivas y de Promoción de la Salud (REDIAPP) (RD06/0018), Instituto Aragonés de Ciencias de la Salud (IACS), Aragón, Spain; 3 Unidad Epidemiología Clínica, Hospital 12 de Octubre, CIBER Epidemiología y Salud Pública, Madrid, Spain; 4 Centro de Salud Arrabal, Zaragoza, Spain; 5 Institut Universitari d'Investigació en Ciències de la Salut (IUNICS), University of Balearic Islands, Palma de Mallorca, Spain; 6 Servicio de Psiquiatría, Hospital Miguel Servet y Universidad de Zaragoza, Zaragoza, Spain; 7 Department of Psychiatry, Miguel Servet Hosìal, Avda Isabel La Catolica 1, 50.009, Zaragoza, Spain

**Keywords:** Fibromyalgia, Memantine, Chronic pain, Magnetic resonance spectroscopy, Randomized controlled trial

## Abstract

**Background:**

Fibromyalgia is a prevalent chronic rheumatic disease of great clinical importance. Recent studies have found raised levels of glutamate in the insula, hippocampus and posterior cingulate cortex regions of the brains of fibromyalgia (FM) patients. This finding has led researchers to speculate about the usefulness of glutamate-blocking drugs such as memantine in the treatment of fibromyalgia. The hypothesis of this study is that the administration of memantine will reduce the glutamate levels, and futhermore, will decrease the perceived pain. The aim of this study is to evaluate the efficacy of memantine in the treatment of pain (pain perception). A secondary objective is to evaluate the efficacy of memantine in the treatment of other clinical symptoms of FM, and to evaluate the efficacy of memantine in reducing brain levels of glutamate, and its effects on the central nervous system as a whole.

**Method/Design:**

A double-blind parallel randomized controlled trial. Participants, Seventy patients diagnosed with FM will be recruited from primary health care centers in Zaragoza, Spain. Intervention. The subjects will be randomized in two groups: A) A treatment group (n = 35), which will receive 20 mg of memantine daily; B) A control group (n = 35), to which will be administered a placebo. There will be a six-month follow-up period (including a titration period of one month). Outcomes. The main efficacy variable of this study is pain (pain perception). The secondary efficacy variables are clinical symptoms (pain threshold, cognitive function, health status, anxiety, depression, clinical impression and quality of life) and glutamate levels in different regions of the brain, which will be assessed by magnetic resonance spectroscopy. Randomization and blinding. Randomization has been computer-generated, and the random allocation sequence will be implemented by telephone. Subjects of the study and the research assistants will be blinded to group assignment.

**Discussion:**

There is a need for the development of innovative and more effective treatments for fibromyalgia. This clinical trial will determine whether memantine can be an effective pharmacological treatment for fibromyalgia patients.

**Trial registration:**

Current Controlled Trials http://ISRCTN45127327 EUDRACT 2011-006244-73

## Background

Fibromyalgia (FM) is a chronic rheumatic disease of currently unknown aetiology that is characterized by the presence of diffuse musculoskeletal pain and painful sensitivity to touch in at least 11 of 18 defined trigger points [1]. The prevalence of this syndrome in Europe is calculated to be approximately 2.9% (95% CI: 2.4 to 3.4) [2]. The prevalence of FM in rheumatology consultations in Spain was found to be 12% (2.2% in men and 15.5% in women) [3]. Because of its high prevalence, there is great clinical impact on patients in terms of disability and loss of quality of life, and significant health care costs. FM treatments are believed to have limited efficacy, with an effect size of approximately 0.5 [4].

Pain is the most common and debilitating symptom of FM. The cause of hyperalgesia in FM is poorly understood. It is suspected that there is an alteration of the function of structures of the central nervous system. In recent years, the neurophysiology of the phenomenon of pain has led to increased interest in employing different neuroimaging methods such as positron emission tomography (PET) [5], single photon emission computed tomography (SPECT) [6,7], functional magnetic resonance imaging (fMRI) and, more recently, magnetic resonance diffusion and diffusion tensor spectroscopy to identify the brain structures activated during episodes of pain in patients and controls [8]. These structures include the primary and secondary sensory and motor cortices, insula, anterior cingulate cortex, thalamus, dorsolateral prefrontal cortex and basal ganglia - regions that have been named the ‘pain matrix’ because they are activated in response to a painful stimulus. A growing body of evidence suggests that glutamate (Glu), an excitatory neurotransmitter in the central nervous system, may play a part in the pathophysiology of FM, given that its concentration is elevated in the insula [8], hippocampus [9] and posterior cingulate cortex [10,11].

As a consequence, a number of authors have suggested that glutamate-blocking drugs may be useful in the treatment of FM [12]. Studies suggest that memantine could reduce the harmful effects that result from the excessively high levels of brain glutamate found in a number of conditions, such as FM [13]. Memantine is not believed to act by reducing levels of glutamate or by preventing its release; rather, it is believed to reduce glutamate’s neurotoxic effect by blocking the N-methyl-D-aspartate (NMDA) receptor, thereby preventing the entry of excess calcium [14]. Memantine belongs to the family of drugs known as NMDA receptor antagonists.

NMDA receptor antagonists have neuroprotective and analgesic properties and are widely used in clinical practice. Dextromethorphan and ketamine have demonstrated efficacy in the treatment of pain in fibromyalgia [15,16], although their use as long-term treatment presents significant difficulties [17]. The NMDA receptor antagonist memantine is a derivative of amantadine, a drug which has been used to treat Parkinson’s disease, spasticity, convulsions, vascular dementia and Alzheimer's disease, and has an excellent clinical safety record spanning more than 20 years. It is a non-competitive open-channel blocker that dissociates from the channel, which allows it to limit the pathological activity of the NMDA receptor without affecting normal synaptic activity [18].

Memantine has shown a very low incidence of side effects in clinical trials on humans [19,20], while a recent extension of trials has demonstrated the drug’s clinical tolerability, even with prolonged use [21]. Similar low rates of adverse effects are expected with the treatment with memantine of other disorders such as pain [22,23], migraine [24] and fibromyalgia [25]. The clinically approved dose of memantine for humans starts with 5 mg/day, increasing progressively over a period of several weeks to 20 mg/day. This progressive dose adjustment may contribute to the drug’s lack of side effects [19]. While this progression reduces the NMDA receptor affinity, it contributes to the safety and efficacy of memantine as a neuroprotective agent. However, the process also makes memantine less effective than high-affinity antagonists (for example, ketamine) in the treatment of chronic pain [21,26]. Nevertheless, recent research has highlighted the efficacy of memantine for the treatment of complex regional pain syndrome [27] and phantom limb pain [28], which suggests that the extent of analgesia depends on the type of pain being treated. We have carried out a preliminary open, uncontrolled, three-month follow-up study of memantine in patients with FM (EudraCT Number: 2011-000802-23). As will be commented later on in the discussion, in this preliminary study there was a trend toward improvement in pain, but it was not significant, probably due to the small sample size. Glutamate levels did not show modifications from baseline as a longer period follow-up is usually required. After three months, memantine-treated patients in our study showed significant improvement in cognitive function, symptoms of depression and global functioning (personal communication).

### Aims

The primary objective is to evaluate the efficacy of memantine in the treatment of pain (pain perception) in patients with fibromyalgia.

The secondary objectives of this study are the following: 1) To evaluate the efficacy of memantine in the treatment of other symptoms of fibromyalgia, such as pain threshold, impaired cognitive function, reduced health status, anxiety, depression, quality of life and perceived improvement of symptoms; 2) To evaluate the efficacy of memantine in reducing brain levels of glutamate, as measured by spectrometry, in patients with fibromyalgia.

## Methods

### Study design

Doubled-blind, multicenter, parallel randomized clinical trial with six-month follow-up.

**Figure 1:**
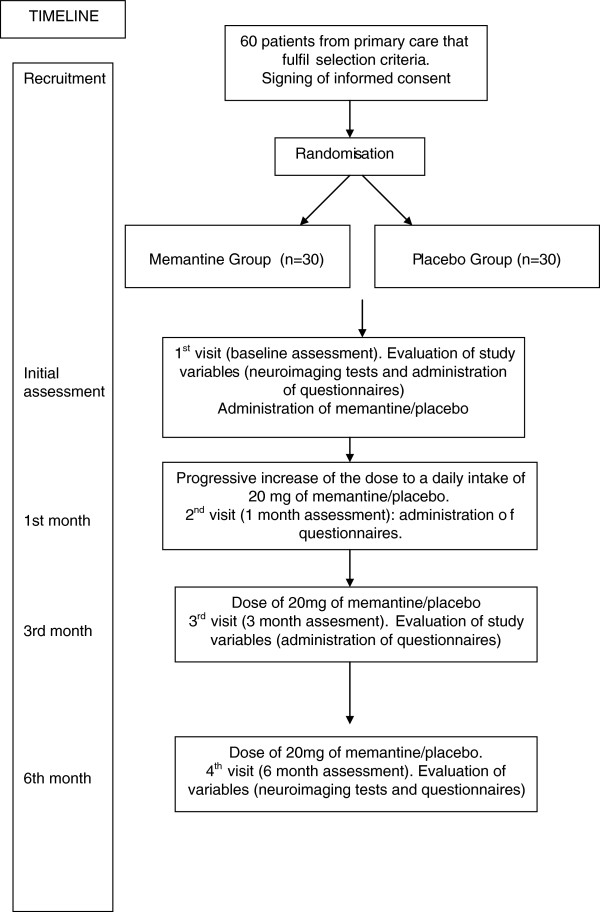
Flowchart of the study.

Seventy patients with FM will be recruited for inclusion in the study upon fulfilment of selection criteria. The patients will be randomized in two parallel groups: a treatment group (n = 35), which will be given 20 mg of memantine daily; a control group (n = 35), which will receive a placebo. There will be a six-month follow-up period (including a dose adjustment period of one month). The study flowchart is shown in Figure 1.

### Setting and study sample (Participants)

Patients diagnosed with FM will be recruited for inclusion in the study from primary health care centers in Zaragoza, Spain, upon fulfilment of the following selection criteria: a) subject (males and females) with age between 18 and 65 years; b) ability to understand Spanish; c) diagnosis of FM performed by a rheumatology specialist according to the American College of Rheumatology (ACR 1990) diagnostic criteria; d) signing of an informed consent form; and e) use of birth control during the study in the case of fertile women.

Patients must not fulfil any of the following criteria at the time of inclusion: a) be undergoing drug treatment for fibromyalgia. In these cases, the patients will discontinue treatment and go through a washout period of one week to minimize the influence of the medication on brain imaging. During that week, the patient may take, if necessary, low doses of analgesics such as tramadol or paracetamol; b) be taking memantine, or have taken memantine during the one year prior to recruitment; c) be suffering from an Axis I psychiatric disorder, as diagnosed using the Structured Clinical Interview for DSM-IV (SCID-I), that might hinder adherence to the protocol (for example, dementia, alcohol and/or substance abuse/dependence, or schizophrenia); d) be pregnant or breast-feeding; e) have a hypersensitivity to the active ingredient memantine, or to the excipients; f) have conditions that require special precautions when administering memantine according to the summary of product characteristics (namely, epilepsy, circumstances that may cause high urinary pH such as *Proteus* urinary infection, renal tubular acidosis or vegetarian diet, recent myocardial infarction, congestive heart disease and uncontrolled arterial hypertension); g) have clinically significant and active evidence of liver or kidney disease, hematological, respiratory, endocrine or cardiovascular disease or disorders. However, patients with controlled diabetes, controlled hypertension and complete or incomplete right bundle branch block can be included in the study; h) use drugs that may cause relevant interactions with memantine according to the summary of product characteristics, namely NMDA receptor antagonists (for example, amantadine, ketamine, dextromethorphan), L-Dopa, dopamine agonists and cholinergic agonists; or i) use non-permitted concomitant medication during the week prior to the first evaluation visit or be expected to require treatment with at least one of the drugs not permitted during the study, namely, antidepressants (for example, duloxetine, venlafaxine, mirtazapine, bupropion, SSRIs, and so on), analgesics (for example, pregabalin, gabapentin, opiates, and so on) or other drugs. During this week, patients may take analgesics such as tramadol or paracetamol if needed, but only sporadically to minimize the influence of the medication on brain images.

There is currently no approved treatment with an indication for the treatment of FM. In 2007, the FDA approved pregbalin as the first drug for the treatment of FM symptoms in the USA. This agency has subsequently approved duloxetine and milnacipran for the same indication. Although these drugs are commercially available in Europe with other indications, European regulatory authorities have recently refused to widen their approval to include them for the treatment of FM [29].

A patient is to withdraw from the trial if he or she withdraws informed consent, if the researcher feels that he or she should withdraw from the study for reasons of safety/efficacy or in the best interest of the patient, or if the patient does not comply with the treatment for more than seven consecutive days.

### Intervention

#### Treatment group

The treatment group will receive the study drug, memantine, in a dose of 20 mg daily for six months, including a one-month titration period.

#### Control group

The control group will receive a daily dose of placebo (coated pills with the same external appearance as the active drug) and will take the same number of pills as the treatment group. Administration will be through the oral route.

This is a double-blind study. Consequently, the patients will be randomized, and neither the patient, the doctor nor the researcher administering questionnaires or spectrometry will know to which group the patient has been assigned. The recommended dose of memantine in adults is 20 mg once a day. To minimize adverse effects, 20 mg doses will be reached by the following titration schema: first week, 5 mg daily; second week, 10 mg daily; third week, 15 mg daily; fourth week, 20 mg daily.

The number of tablets in each dispensed container will be monitored at each evaluation, keeping track of the number the patient should have taken and how many should remain on completion of the treatment. The route of administration is oral, in the form of film-coated tablets. The drug for use in the study (both memantine pills and placebo) will be prepared, conditioned and released by one qualified person according to the principles of Good Manufacturing Practice, under the responsibility of H Lundbeck A/S. Upon completion of the trial, patients will continue with standard FM treatment according to clinical practice guidelines.

### Outcomes and measurements

#### Main outcome variables

The main efficacy variable is the improvement in the treatment of pain, specifically pain perception. Perceived pain will be measured by means of a visual analogue scale (VAS) from 0 to 100. The psychometric usefulness of VAS has been widely demonstrated [30].

#### Secondary variables

Secondary efficacy variables are modifications made to values of the following clinical variables: pain threshold, cognitive state, health status, state of anxiety and depression, clinical improvement impression and quality of life.

Pain threshold will be measured by means of a sphygmomanometer, a widely used clinical test that has been demonstrated to be useful for identifying FM patients [31]. It is recommended that the blood pressure cuff should be inflated in increments of approximately 10 mmHg up to 180 mmHg or to the point that pain appears. Healthy persons tend to feel pain when the pressure cuff is inflated to 160 mmHg or more, while FM patients generally present pain at pressures between 100 and 110 mmHg, or even lower. Blood pressure should be recorded to adjust for the effect of hypertension on pain threshold.

The rest of the secondary efficacy variables will be measured by structured exams. The Cognition Mini-Exam (MEC) will measure cognitive state. The MEC is a structured scale that consists of 35 points grouped into seven categories: orientation to place, orientation to time, recall, attention and concentration, memory, language and visual construction. In non-geriatric populations (under the age of 65) such as the sample for this study, the threshold that suggests a ‘likely case’ of a cognitive disorder is 27 points and lower. This test is the validated Spanish-language version of the Mini-Mental State Examination (MMSE) [32].

Health status in FM will be measured by means of the Fibromyalgia Impact Questionnaire (FIQ). The FIQ is a ten-item self-assessment questionnaire that measures the health status of FM patients. The validated Spanish-language version of this questionnaire will be used [33]. Anxiety and depression will be measured by means of the Hospital Anxiety Depression Scale (HADS). HADS is a scale based on self-report that was developed to detect the presence of depression and anxiety disorders in medical patients in primary care settings. It contains fourteen items scored on a four-point Likert-type scale. This scale is comprised of two subscales that separately assess depression and anxiety. The validated Spanish-language version of this scale will be used [34]. Quality of life will be measured by means of the EuroQol 5D (EQ5D) questionnaire. This questionnaire is a standardized instrument used as a measure of health outcomes. It is applied to a wide range of health conditions and treatments and provides a simple descriptive profile and a single index value for health status. The Spanish-language version of this questionnaire will be used [35]. Perceived clinical improvement will be assessed by the Clinical Global Impression scale (CGI).

#### Magnetic resonance spectroscopy

Another relevant secondary efficacy variable in this study is the level of glutamate in different regions of the brain, namely the insula, hippocampus and posterior cingulate cortex, as assessed by magnetic resonance spectroscopy (MRS).

The following parameters will used for Magnetic Resonance Spectroscopy (MRS): a T2-weighted coronal image with repetition time (TR) = 5,350 ms, echo delay time (ET) = 85 ms, 90° flip angle, number of excitations = 2, imaging matrix = 320 × 256, field of view (FOV) = 24 cm × 24 cm and slice thickness/distance = 5/0 mm and taken on the plane through the internal auditory canal and cerebral peduncle will be used to locate volumes of interest (VOIs) (2 × 2 × 2 cm) in both hippocampi. A T1-weighted medial sagittal image (TR = 560 ms, ET = 12 ms, 90° flip angle, number of excitations = 1, imaging matrix = 256 × 160, FOV = 24 cm × 24 cm, slice thickness/distance = 5/0 mm) will be obtained to locate a voxel in the posterior cingulate cortex, and a parasagittal T1-weighted image will be taken with respect to the right of the corpus callosum plane to locate voxels on the anterior and posterior insular regions. Proton magnetic resonance spectroscopy will be performed with a short ET of 35 ms, a TR of 2,000 ms and 128 accumulations by means of the single-voxel spin-echo technique, which uses selective excitation with gradient spoiling for water suppression. Spectral acquisition will be accomplished by means of the PROBE/PRESS (proton brain spectroscopy/point-resolved spatially localized spectroscopy) technique. LCModel (version 6.0) user-independent frequency domain-fitting software will be used to quantify absolute concentrations of brain metabolites in mmol/kg. Eddy current correction will be applied, and the use of the internal water signal will be the reference from which the absolute metabolite concentrations will be calculated. In addition to the individual analysis of glutamate (Glu), creatine (Cr), N-acetylaspartate (NAA) and myo-inositol (mI) compounds, the aggregate concentrations of the following three compound pairs will be studied: NAA + N-acetyl-aspartyl-glutamate (NAA + NAAG), referred to as the total phosphocholine (PCh); Glycerophosphorylcholine (GPC), referred to as the total Cho; and glutamate + glutamine, referred to as Glx. Absolute metabolite concentrations will be considered only if the Cramér-Rao lower bounds are lower than 20%, indicating that these metabolite concentrations can be reliably calculated. Chemical concentrations can be extracted automatically from MR spectra with well-documented time domains, and the spectral frequency can be fitted with software packages such as LCModel (Stephen Provencher, Oakville, Ontario, Canada).

Table 1 shows the procedure and evaluation times of the trial.

**Table 1:** Procedure and evaluation times of the trial

**Visit**	**Baseline**	**1 month**	**3 months**	**6 months**
Visit number	1	2	3	4
**Recruitment & Clinical assessment**				
Signing of informed consent	X			
Diagnosis	X			
Inclusion/exclusion criteria	X			
Sociodemographic variables	X			
Pain assessed with sphygmomanometer	X	X	X	X
Pain assessed with Analogue Visual Scale	X	X	X	X
Cognitive function (assessed with MEC)	X	X	X	X
Global function (evaluated by FIQ)	X	X	X	X
Anxiety and depression (assessed with HADS)	X	X	X	X
Quality of life (assessed by EuroQol 5D)	X	X	X	X
Clinical Global Impression (CGI)	X	X	X	X
**Neuroimage assessment**				
Magnetic resonance spectroscopy	X			X
**Safety assessments**				
Adverse events		X	X	X
**Other study procedures**				
Delivery of the research drug	X	X	X	
Inventory of drug taken back and used		X	X	X
Concomitant pharmacological treatment	X	X	X	X

### Sample size

The choice was made to have a sample comprising 70 subjects (n = 35 subjects in each of the two treatment groups) because this sample size will enable us to obtain reliable results and to ensure that both treatment groups are of equal sizes at the start of the study. Smaller sample sizes have been used in the identification of significant differences in glutamate levels in different brain regions between FM patients and controls.

Using the results of the pilot study and taking reduction of pain (as determined using the Pain Visual Analogue Scale and sphygmomanometry) as the primary variables, a pre-intervention mean score of 56 (SD 14.9) was obtained with the VAS, and 104 (SD 30.8) with sphygmomanometry. After treatment, the means decreased to 44 (SD 16.9) and 85 (SD 20.6), respectively. Therefore, assuming a 95% confidence interval and a power of 80%, we find that we require a sample size of 28 individuals in each group for the VAS finding and of 30 individuals in each group for the reduction measured by sphygmomanometry. The resulting sample size that will enable us to analyse the final variable will be 60 individuals, with 30 being assigned to each of the two groups. Based on previous studies (personal communication), an attrition rate of 10% can be expected. Therefore, the final number of recruited patients will be 35 patients for each arm.

### Randomization, allocation and masking of study groups

Each patient will be assigned to one of the two groups using a computer-generated random number sequence. The assignment will be carried out by an independent person belonging to REDIAPP (Research Network on Preventative Activities and Health Promotion) who is not involved in the study. The random allocation sequence will be implemented by telephone. The sequence will be concealed until interventions are assigned. Patients agree to participate before the random allocation and without knowing to which treatment they will be assigned. According to the intention-to-treat (ITT) principle, any patients randomized and allocated to any arm that starts the study will be included in the analysis. Pharmacological treatment will be administered by one psychiatrist (JGC). Study personnel conducting psychological assessments (BO, MCPY, MP, BM) will be masked to the participants' treatment.

### Statistical methods

#### Analysis strategy

The analysis of clinical efficacy will make use of intention-to-treat analysis. The Last-Observation-Carried-Forward (LOCF) method will be used for handling missing data. An initial comparison will be made between both groups, examining key variables to establish the groups’ baseline comparability after randomization. To describe the quantitative variables, means and standard deviations will be calculated when they fulfil normality criteria. The Chi-squared test will be used for qualitative variables such as a number of socio-demographic ones. The differences between clinical variables at baseline, one month, three months and six months will be calculated using analysis of covariance (ANCOVA) adjusted by baseline.

To study the main variable, analysis of variance will be performed on repeated measurements, including all the evaluations over time. The main variable (pain perception) will be taken to be a continuous variable for this purpose. The models will include adjustments for the baseline pain value and for any other variable that may have shown differences in the baseline measurement. Possible group × time interactions will be studied by means of Mixed Factor ANOVA. Additionally, linear regression models will be used to compare the differences between the two groups for each of the evaluations over time as compared to baseline. Similar analyses will be performed on the other secondary clinical variables.

To analyze the differences found by the neuroimaging test (spectrometry) taken at baseline and at six months follow-up, Student’s *t*-test for paired measurements will be calculated. The relation between neuroimaging variables and clinical variables will be assessed by means of Spearman’s Rho non-parametric test.

In case of lost values, a sensitivity analysis will be made to estimate the effect of the lost values on the results. These values will also be replaced using several approaches, such as last recorded values and imputations. Statistical analyses will be performed with the Statistical Package for the Social Sciences (SPSS) 19.0 statistical software package (SPSS Inc., 233 South Wacker Drive, 11th Floor, Chicago, IL 60606–6412), with *P-*values below 0.05 considered significant.

### Safety and monitoring

Any adverse events and serious adverse events will be recorded and a determination will be made as to whether any medical action required is related with the administered drug. Any adverse event related to the study drug will be recorded and monitored until its resolution or stabilization, and the regulatory notification process will be followed in accordance with the pertinent legislation in force (timeframes, unmasking, and so on). Study participants will be requested to report to the researchers any adverse effect (serious or otherwise) that may arise in the period between visits or any other circumstances that may lead to their withdrawal from the study.

### Ethical aspects

Informed consent will be obtained from the participants before they are aware of which group they are to be included in. Before they give their consent, the patients will be provided with a general overview of the aims and characteristics of the study and the psychological and pharmacological intervention. They will also be informed that they will be participating voluntarily and that they can choose to withdraw at any time with the guarantee that they will continue to receive the treatment considered most appropriate by their doctor.

With regard to the potential risks of the study, data gathering involves no risks to the subjects participating in the study, and the neuroimaging techniques that will be performed are non-invasive techniques that do not place subjects in any danger. With regard to the potential benefits, if the hypothesis of the study, the patients in the treatment group will be able to control their pain during the course of this research. The results will be communicated to the scientific community and to the relevant institutions, which will lead to the expected benefits being obtained for society and for people diagnosed with FM. Nevertheless, there is a possibility that there will be no benefit gained from participating in this study.

The study follows Helsinki Convention norms with posterior modifications, and the Declaration of Madrid of the World Psychiatric Association. The Study Protocol was approved by the Clinical Research Ethics Committee of Aragón (June 2012) and the Medicines and Health Products Agency of Spain (EUDRACT 2011-006244-73).

## Discussion

This is the first randomized, controlled study of memantine for the treatment of fibromyalgia. Memantine might be expected to be useful for the treatment of FM based on its pharmacological effects, as described in the introduction [13,14]. In addition, our research group have recently carried out an exploratory, uncontrolled study with a small sample (n = 10) and a three-month follow-up that found significant improvement of global function (as measured by FIQ), depression (as evaluated by HADS) and cognitive function (as assessed with MEC) in the study patients at three-month follow-up (personal communication, Study EudraCT Number: 2011-000802-23). However, at three-month follow-up, glutamate levels did not decrease in any of the brain areas studied (personal communication).

Based on previous preliminary pilot studies (personal communication) a significant improvement in key clinical symptoms in fibromyalgia, such as depression, cognition and global function can be expected. Cognition is one of the most impairing symptoms in fibromyalgia [36] and is strongly correlated with depression [37]. Cognition in these patients seems to be related to brain morphology changes that could be improved with memantine [38]. Depression, another frequent and quite disabling symptom in fibromyalgia [39] is also expected to improve with memantine due to its pharmacological effect [40].

Pain, the most relevant symptom in fibromyalgia and the main outcome of our study, was not significantly relieved in our open, uncontrolled study (personal communication), probably due to its small sample size. However, previous pilot studies (26 to 28) suggest memantine is effective in relieving pain in general. In addition, spectrometric studies in fibromyalgia found a strong correlation between high levels of glutamate and depression and global function, but also with pain (10).

Treatment with memantine decreases the Glu/Cr (glutamate/creatine) ratio in the left hippocampal region, which may account for its anti-excitotoxic property [41]. Other studies have observed an increase in cingulate glutamatergic turnover [42]. There is evidence from MRS studies of other excitotoxicity-related conditions, such as amyotrophic lateral sclerosis [43] and Huntington’s disease [44], that glutamate is elevated in several brain regions in these disorders. Thus, the decrease in hippocampal glutamate with memantine (without changes in NAA/Cr) could explain some of memantine’s therapeutic effects.

In this study, there is no clear hypothesis as to whether memantine is able to reduce glutamate levels in the affected brain areas, or, independent of this possibility, whether the drug will be an effective treatment for different clinical symptoms of fibromyalgia, particularly in the cognitive domain. It is possible that it could reduce glutamate without ameliorating any symptoms. Alternatively, it may not reduce glutamate while improving some target symptoms. In any case, new hypotheses as to the aetiology of fibromyalgia, and of the origins of the symptoms of the disease may be developed based on the results of this preliminary research.

The main strength of the study is that this is the first randomized controlled trial of memantine for the treatment of FM. The main limitations specific to the spectrometric study will be the following: 1) The proportion of grey matter/white matter and CSF within the corresponding voxel will not be calculated, so the 1H-MRS signals, which are produced by both grey and white matter, will represent an amalgamation of multiple cell types. Differences in the fractions of each voxel consisting of different tissue types may result from anatomical differences associated with the conditions under investigation. 2) Metabolites will not be quantified by segmentation and CSF-correction, so the measured levels of glutamate will often be contaminated in part by glutamine. Furthermore, glutamate exists in the neurotransmitter pool as well as in the metabolic pool.

## Trial status

Inclusion of patients commenced 15 September 2012.

Initial recruitment of patients commenced 15 September 2012.

Expected finalization of patient recruitment: 31 December 2012.

Expected finalization of patient monitoring period: 30 June 2013.

Publication of results: December 2013.

## Abbreviations

ACR: American College of Rheumatology;ANOVA: Analysis of variance;CGI: Clinical Global Impression;CI: Confidence interval;Cr: Creatine;EQ5D: EuroQol 5D;ET: Echo delay time;FIQ: Fibromyalgia Impact Questionnaire;FM: Fibromyalgia;fMRI: Functional magnetic resonance imaging;FOV: Field of view;Glu: Glutamate;GPC: Glycerophosphorylcholine;HADS: Hospital Anxiety Depression Scale;MEC: Cognition Mini-Exam;LOCF: Last-Observation-Carried-Forward;mI: myo-inositol;MMSE: Mini-Mental State Examination;MRS: Magnetic resonance spectroscopy;NAA: N-acetylaspartate;NAAG: N-acetyl-aspartyl-glutamate;NMDA: N-methyl-D-aspartate;PCh: Phosphocholine;PET: Positron emission tomography;PROBE/PRESS: Proton brain spectroscopy/point-resolved spatially localized spectroscopy;REDIAPP: Research Network on Preventative Activities and Health Promotion;SD: Standard deviation;SCID-I: Structured Clinical Interview for DSM-IV axis-I;SPECT: Single photon emission computed tomography;TR: Repetition time;VAS: Visual analogue scale;VOIs: Volumes of interest

## Competing interests

The authors declare that they have no conflicts of interest. The research group that designed and developed this study is financed by the Department of Science, Technology and University of the Government of Aragon and by the Carlos III Institute of Health, which is attached to the Spanish Ministry of Science and Innovation.

## Authors´ contributions

JGC, RM, BO, MR, YLdH and ASB are the principal researchers and developed the original idea for the study. The study design was further developed by MCPY, MP and BM. EA will develop the statistical methods. All authors have read and corrected draft versions and approved the final version.
